# No more government-imposed societal-level COVID-19 control measures but still significant self-experienced burden for severely immunocompromised individuals – A cross-sectional survey in the Netherlands

**DOI:** 10.1016/j.pmedr.2024.102827

**Published:** 2024-07-14

**Authors:** Jan Pander, Wendy Beekman-Hendriks, Neeltje Coolen, Valerie van de Flier, Jeroen Senster, Chantal P. Rovers

**Affiliations:** a AstraZeneca BV, PO Box 93015, 2509AA Den Haag, the Netherlands; b Vergeet Ons Niet VWS, the Netherlands; c Motivaction BV, PO Box 15262, 1001MG Amsterdam, the Netherlands; d Radboud University Medical Center, Department of Internal Medicine/Division of Infectious Diseases, PO Box 9101, 6500HB Nijmegen, the Netherlands

**Keywords:** COVID-19, Immunocompromised, General well-being, Mental health, Physical health, Daily activities, Social activities, Government-imposed societal measures, Self-isolation, Shielding

## Abstract

**Objectives:**

In March 2023, all societal-level COVID-19 control measures were lifted by the Dutch government. This study was performed to understand the self-experienced burden of this new phase of COVID-19 on the perspectives and behaviors of severely immunocompromised individuals.

**Methods:**

This is an observational, descriptive, cross-sectional study in The Netherlands. An online survey was completed by severely immunocompromised individuals, to capture their general well-being (score from 1 = worst to 10 = best), mental and physical health, and daily and social activities during survey conduct and retrospectively for before onset of COVID-19. The survey was open for completion from May 24th until August 7nd, 2023.

**Results:**

Of the 236 respondents, 96.6 % had been vaccinated against COVID-19 and 24.6 % were shielding to avoid COVID-19 during survey conduct. The general well-being score for all respondents was 7.5 (±1.2 SD) before onset of the COVID-19 pandemic and 6.9 (±1.6 SD) during survey conduct (P<0.001). For the shielding group (n = 58), these scores were 7.6 (±1.0 SD) and 5.7 (±1.6 SD), respectively (P<0.001). Generally, for all questions about mental and physical health and daily and social activities, there was a trend towards more negative answers during survey conduct, compared with before onset of the COVID-19 pandemic, which was more pronounced for the shielding group.

**Conclusions:**

Despite absence of government-imposed societal measures, COVID-19 avoidance still had a self-experienced burden on perspectives and behaviors of immunocompromised individuals in The Netherlands, with a significantly lower general well-being during survey conduct, compared with before onset of COVID-19.

## Highlights

• 236 Dutch immunocompromised people completed a survey on avoiding COVID-19.
• The survey was conducted after national COVID-19 control measures were lifted.
• 1 out of 4 immunocompromised individuals were still shielding to avoid COVID-19.
• Self-reported general well-being was significantly lower than before COVID-19.

## 1 Introduction

Coronavirus disease 2019 (COVID-19) was declared a pandemic by the World Health Organization (WHO) in March 2020 (WHO, 2023a). As of March 2024, more than 774 million confirmed COVID-19 cases had been reported globally, resulting in more than 7 million reported deaths (WHO, 2024).

A major concern early in the pandemic was that the demand for health care services exceeded capacity, especially on intensive care units (ICU). To mitigate this, the Dutch government deployed societal measures to prevent this and to protect the population at high-risk for severe COVID-19 (RIVM, 2022b). These measures were aimed at minimizing physical contact and close-proximity exposures between individuals, and included closure of schools, shops, pubs, clubs and restaurants; working from home; 1.5 m physical distancing; restrictions on public and private gatherings; wearing face masks; and temporarily even a curfew. The national COVID-19 vaccination program started in January 2021 in the Netherlands – and immunocompromised patients were invited for vaccination by the end of March 2021. Approximately 82 % of the Dutch adult population completed at least the primary vaccination series (RIVM, 2023a). However, COVID-19 was and remains a threat for immunocompromised persons, in part due to their suboptimal responses to vaccines (Evans et al., 2023, Shoham et al., 2023).

In March 2023, all societal COVID-19 prevention measures were lifted by the Dutch government (DUTCH GOVERNMENT, 2023). This was based on the success of the vaccination campaign, the low number of COVID-19 related hospital/ICU admissions, and the reduced severity of the Omicron variant compared to earlier variants (Hyams et al., 2023, Jassat et al., 2022). Even though COVID-19 is no longer a Public Health Emergency of International Concern in the perspective of the WHO, it was still considered a pandemic until two months after the Dutch measures were lifted (WHO, 2023b).

The end of the government-imposed societal measures was a relief for the majority of the Dutch population. However, individuals with a compromised immune system – due to certain medical conditions and/or the use of immunosuppressive medication(s) (RIVM, 2023d) – still have considerably increased risks for COVID-19 and related hospitalizations, ICU admissions, and death compared with those of the general population (Evans et al., 2023, Shoham et al., 2023). This is likely due to a lower, insufficient vaccination response from their impaired immune system (Lee et al., 2022, Tartof et al., 2022). For this reason, immunocompromised individuals and their household members may still feel the necessity to voluntarily minimize or eliminate physical and close-proximity interactions with others to protect themselves against severe COVID-19 – which may in turn have unintended negative impacts on their general well-being and health (Antoun et al., 2021, Westcott et al., 2021).

It is likely that immunocompromised individuals and their household members also display such shielding behavior in The Netherlands, while for the rest of society COVID-19 no longer has a noticeable impact. The behavior of – and the resulting impact on – immunocompromised individuals is currently not described in detail and awareness of this issue is limited. For policy makers and care givers it is therefore crucial to have deeper insights into the behavior and well-being of immunocompromised individuals in this phase of COVID-19 to determine whether (and which) specific preventive measures and/or care are needed – because currently no specific measures other than limited possible COVID-19 booster vaccinations are implemented to protect this vulnerable group.

The primary objective of our study was therefore to describe the self-experienced burden of COVID-19 avoidance of severely immunocompromised individuals, during a period when no more government-imposed societal measures for COVID-19 prevention were in place.

## 2 Methods

### 2.1 Study design and survey development

This is an observational, descriptive, cross-sectional study in The Netherlands. A new online survey was developed with study specific questions, to capture the perspectives and behaviors of severely immunocompromised individuals during survey conduct and retrospectively for before onset of the COVID-19 pandemic. The survey questionnaire was developed with input from patient-organizations representing the target population (National MS Fund; National Association for Lupus, Anti-phospholipid Syndrome, Scleroderma and Mixed Connective Tissue Disease (NVLE); Foundation for Immune Disorders (SAS); Dutch Kidney Patients Association (NVN); Heart and Lung Transplant Association; Vergeet ons niet VWS), though it was not formally validated for this study. The survey questionnaire included questions about general well-being, mental and physical health, daily and social activities, work and individual protective measures. It also contained questions about the well-being of their household members. General well-being was scored on a numerical scale from 1 (worst) to 10 (best). For questions about mental and physical health and daily and social activities, the responses were obtained on a 5-point ordinal scale ranging from ‘always’, through ‘often’, ‘sometimes’, and ‘rarely’, to ‘never’, and included ‘not applicable’. Because it was technically not possible to skip questions in this digital questionnaire, there were no missing data.

The online platform for the survey was developed and hosted by Motivaction BV (Amsterdam, The Netherlands), and was administered as a non-personalized web-link for participants’ self-administration. The survey questionnaire contained no open text fields, and the obtained responses were untraceable to individuals and therefore completely anonymous. Due to this methodology, multiple administrations per individual were possible, but this was considered unlikely.

### 2.2 Data collection

The survey was open for completion from May 24, 2023 until August 7, 2023, which was two to five months after government-imposed, societal-level COVID-19 measures had been lifted in the Netherlands, when the COVID-19 Omicron variant was circulating (DUTCH GOVERNMENT, 2023). Also, no preventive therapies other than vaccinations were available in the Netherlands during this period. Participants were asked at this one time point to answer questions about their situation for two time periods: for the current time period of over the past 2 weeks (during survey conduct) and retrospectively for the time period of just before onset of the COVID-19 pandemic (i.e., at the end of 2019).

### 2.3 Study population

Inclusion criteria for the study were:

- Age 18 years or older;
- Able to read and understand Dutch;
- Willing to complete an online survey;
- Self-reported condition associated with a severely compromised immune system, which is one of the following groups for whom booster COVID-19 vaccinations are recommended by the Dutch National Institute for Public Health and the Environment (Rijksinstituut voor Volksgezondheid en Milieu; RIVM) (RIVM, 2023d):
  o Solid organ or stem-cell transplantation;
  o Dialysis;
  o Primary immunodeficiency disorder;
  o Active hematological malignancy;
  o The treating physician indicated the immune system was compromised due to a medical condition or receipt of immunosuppressive treatments.

Exclusion criteria were:

- Currently hospitalized individuals;
- Onset of immunocompromised condition after the start of the COVID-19 pandemic.

Participants for this study were recruited via patient advocacy groups representing patients with a condition associated with a severely compromised immune system. Screening questions were included in the survey to ensure inclusion of only the eligible study population. The survey could not further be completed when respondents were ineligible as a result of the screening questions.

### 2.4 Ethical approval

The study was performed in accordance with ethical principles consistent with the Declaration of Helsinki, ICH GCP, and the applicable legislation on non-interventional studies in The Netherlands. The study protocol was independently registered at, and approved by the Dutch Clinical Research Foundation (DCRF; nWMO23.03.003/MEC number 2023-063) according to the Dutch assessment framework for observational studies (www.nwmostudies.nl). Since the survey was completely anonymous, a signed informed consent was not needed per the DCRF and was therefore not obtained. No financial compensation was given to the respondents. The study was funded by AstraZeneca BV, The Netherlands.

### 2.5 Outcomes

The primary outcome measures included the responses to questions about COVID-19 avoidance (i.e., “shielding”), general well-being, mental and physical health, and daily and social activities. Respondents who answered ‘always’ or ‘often’ to at least 3 out of 5 of the questions about staying away from (1) family and friends, (2) bars, restaurants, theatres or cinemas, (3) sport clubs, gym or hobby clubs, (4) work and/or school or (5) public transport during survey conduct were considered to be shielding themselves and thus defined to be in the “shielding group”. In the case the answer to one or more of these questions was ‘not applicable’, at least 3 out of 4, 2 out of 3, 2 out of 2, or 1 out of 1 questions had to be answered with 'always' or 'often' in order to be considered as shielding and thus defined to be in the “shielding group”.

Additional outcomes of the study included responses to questions about self-experienced burden of household members, work, and individual protective measures.

### 2.6 Statistical analysis

The primary objective of this study was to describe the self-experienced burden of COVID-19 avoidance on the perspectives and behaviors of severely immunocompromised individuals, during a period when no more government-imposed societal measures for COVID-19 prevention were in place. Even though no formal sample size calculation was necessary for this descriptive-only study without an a priory hypothesis, a sample size of 200 to 300 respondents was considered acceptable for a maximum inaccuracy of measurement of 6.9 % at a 95 percent confidence level.

The following statistical comparisons were performed to aid the interpretation of findings: the mean score for general well-being for the time period of before onset of the COVID-19 pandemic (i.e., at the end of 2019), though obtained during survey conduct, was compared with the mean score for general well-being for the time period of during survey conduct using a paired Student’s *t*-test, both in the entire study population as well as for the shielding only group separately. Also, the mean score for general well-being during survey conduct of the shielding group was compared with the non-shielding group with a non-paired Student’s *t*-test. Histograms of the data were visually inspected and confirmed for normal distribution. No other statistical comparisons were made. A p-value of < 0.05 was considered statistically significant. No adjustments were made for multiple testing.

## 3 Results

### 3.1 Baseline characteristics

A total of 274 individuals completed the survey, of whom 236 were included in the analysis because their severely immunocompromised condition was already present before onset of the COVID-19 pandemic (i.e., at the end of 2019) and they met all other enrollment criteria. The baseline characteristics of the study population is shown in Table 1. Among the study population, almost all (96.6 %) had been vaccinated against COVID-19, and 58 (24.6 %) of the study population were shielding themselves during survey conduct with the remaining 178 (75.4 %) being non-shielding.

**Table 1:** Baseline characteristics of the study population of severely immunocompromised individuals in The Netherlands during survey conduct (May 24, 2023–August 7, 2023); n = 236.

Characteristic	Value	N	%
Gender	Male	73	30.9
	Female	162	68.6
	Other	1	0.4
Age	18–25 years	4	1.7
	26–35 years	15	6.4
	36–45 years	33	14.0
	46–55 years	50	21.2
	56–65 years	66	28.0
	>65 years	68	28.8
Previous COVID-19 disease	Yes	176	74.6
	No	60	25.4
Vaccinated for COVID-19*	Yes	228	96.6
	No	8	3.4
Reason(s) for severely immunocompromised status**	Active hematological malignancy	4	1.7
	Solid organ or stem-cell transplantation	151	64.0
	Dialysis	2	0.8
	Primary immunodeficiency disorder	24	10.2
	Other***	91	38.6
Household composition at the time of the survey	Living alone	42	17.8
	Living with parent(s)	4	1.7
	Living with child(ren) (no partner)	5	2.2
	Living with child(ren) and partner	49	20.8
	Living with partner only	132	55.9
	Other	4	1.7
Shielding during survey conduct****	Yes	58	24.6
	No	178	75.4

* Number of vaccinations was not reported.

** Multiple answers were possible; therefore the sum of the percentages exceeds 100%.

*** Treating physician had indicated their immune system was compromised due to a medical condition or receipt of immunosuppressive treatments.

**** Respondents who answered ‘always’ or ‘often’ on at least 60% of the questions about staying away from (1) family and friends, (2) bars, restaurants, theatres or cinemas, (3) sport clubs, gym or hobby clubs, (4) work and/or school or (5) public transport at the time of completing the survey were considered shielding themselves.

### 3.2 Self-experienced general well-being

The mean scores for general well-being are shown in Table 2. For the entire study population these scores were retrospectively reported to have been 7.5 (±1.2 SD) before onset of the COVID-19 pandemic (i.e., at the end of 2019) and 6.9 (±1.6 SD) reported during survey conduct, a statistically significant difference (P < 0.001). For the 58 respondents who were shielding themselves during survey conduct, the mean score for general well-being was 7.6 (±1.0 SD) before the onset of the COVID-19 pandemic and 5.7 (±1.6 SD) during survey conduct, a statistically significant difference (P < 0.001). In addition, the mean score for general well-being during survey conduct was lower for shielding individuals compared with the non-shielding portion of the study population, with a statistically significant difference (5.7 versus 7.3, respectively; P < 0.001).

**Table 2:** Scores for general well-being of severely immunocompromised individuals before onset of the COVID-19 pandemic (at the end of 2019) and during study conduct (May 24, 2023 until August 7, 2023) in The Netherlands.

	n	Before onset of the COVID-19 pandemic (at the end of 2019)	During study conduct	P value
Entire study population	236	7.5 ± 1.2 SD	6.9 ± 1.6 SD	P < 0.001
Shielding population	58	7.6 ± 1.0 SD	5.7 ± 1.6 SD	P < 0.001
Non-shielding population	178	7.4 ± 1.3 SD	7.3 ± 1.4 SD	P = 0.06

### 3.3 Self-reported mental health, physical health, daily activities and social activities

**Fig. 1:**
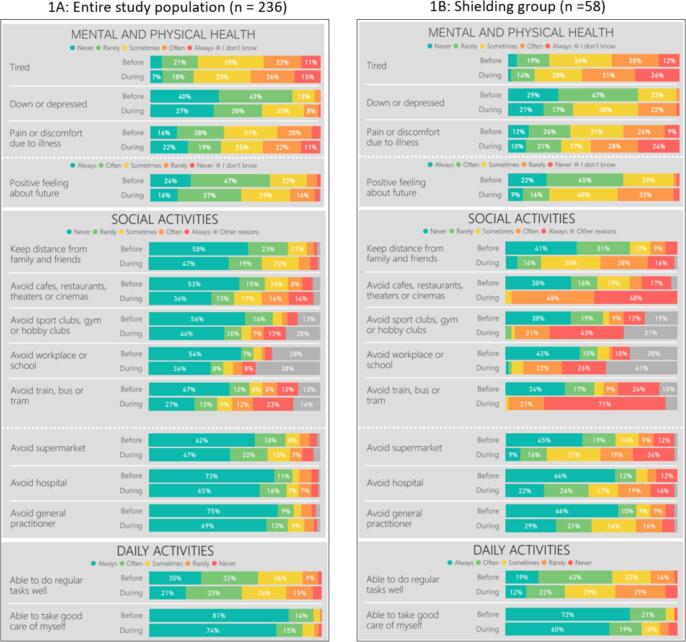
Responses to questions about mental and physical health and daily and social activities, before onset of the COVID-19 pandemic (i.e., at the end of 2019) versus during the survey collection period (May 24, 2023 – August 7, 2023) in The Netherlands, for A: the entire study population of all severely immunocompromised individuals (n = 236) and B: the shielding group (n = 58).

The responses to questions about mental and physical health, daily activities, and social activities for the entire study population (n = 236) and the shielding group (n = 58) are shown in Fig. 1A and 1B, respectively. Even though no statistical tests were performed on these data, for all questions there was a trend to more negative perceptions and behaviors during survey conduct compared with before onset of the COVID-19 pandemic (i.e., at the end of 2019) (Fig. 1A). This informally observed trend was more pronounced for the shielding group (Fig. 1B) than for the entire study population. In the shielding group, 33 % indicated to often or always avoid hospital visits during survey conduct.

### 3.4 Self-experienced burden on household members, work and individual measures

The responses to questions about participant observer-reported behaviors of household members (for the 194 respondents not living alone) for before the COVID-19 pandemic (i.e., at the end of 2019) versus during survey conduct were generally similar, except for household members feeling tired (data not shown).

Of the 130 (55.1 %) respondents who were working (both paid as well as volunteer work) during survey conduct, 54 (41.5 %) reported an influence of the pandemic on their work behavior, of which the majority (n = 34; 26.2 %) was working more from home while 9 (6.9 %) quit their jobs and 6 (4.6 %) were required to be physically present against their will.

Despite the absence of government-imposed societal COVID-19 control measures at the time of the survey, 129 respondents (54.7 %) reported still taking individual protective measures, including wearing face masks (n = 55; 23.3 %), engaging in less physical contact in social situations (n = 54; 22.9 %), and staying home as much as possible (n = 37; 15.7 %).

## 4 Discussion and conclusion

In this study we showed that the general self-experienced well-being of severely immunocompromised individuals in The Netherlands was lower during the COVID-19 Omicron period when no more government-imposed societal-level COVID-19 control measures were in place – compared with their self-experienced general well-being before onset of the COVID-19 pandemic (i.e., at the end of 2019). This difference was more pronounced for the 24.6 % of the study population who reported that they were shielding to avoid COVID-19 and met our definition of shielding. Also, there was a difference on multiple dimensions of self-experienced and self-reported mental and physical health and daily and social activities during survey conduct compared with before onset of the COVID-19 pandemic (i.e., at the end of 2019).

Until September 2022, the Dutch National Institute for Public Health and the Environment (RIVM) conducted population-based cohort research on well-being of the general Dutch population. Over the course of the COVID-19 pandemic, the average score for well-being in that research varied between 6.9 and 7.6, but specific data for severely immunocompromised individuals in the population were not reported (RIVM, 2022a). Recently presented surveys from the RIVM (October 2023) indicated that immunocompromised individuals are more worried about COVID-19 (RIVM, 2023b), which is in line with our findings.

Research in other countries and other populations also showed similar findings. People with cystic fibrosis in the United Kingdom, who were advised to stay at home and avoid contacts early in the COVID-19 pandemic, reported significantly increased anxiety scores during a 4-month shielding period (Westcott et al., 2021). Similarly, COVID-19 restrictions were found to be negatively associated with physical activity, well-being and quality of life in end-stage renal disease patients in the United Kingdom (Antoun et al., 2021). Very recently presented research also showed that about two-thirds of immunocompromised adults in the US and UK continued to physically distance themselves to avoid COVID-19 after societal-level measures were lifted – and this behavior was associated with impaired quality of life (Lloyd et al., 2023, Powell et al., 2023). Also, a recently presented survey from the universities of Liverpool and Bath showed that immunocompromised individuals in the UK were feeling more worried and more depressed during the post government imposed restrictions period compared with the general population in the UK (Bernardi and Daniels, 2023). A comparison of our findings (i.e. behavior and well-being after restrictions were lifted) with other vulnerable groups such as elderly and people with cancer would be of interest, but to our knowledge this has not yet been investigated.

The strengths of our study include that the study population was a heterogeneous sample of severely immunocompromised individuals in the Dutch population. Severely immunocompromised individuals are an important target group for booster COVID-19 vaccinations, as indicated by the Dutch National Institute for Public Health and the Environment (RIVM) (RIVM, 2023c), and the number of such individuals are estimated to be around 200,000–400,000 in The Netherlands (DUTCH FEDERATION FOR NEPHROLOGY, 2021). Also, we investigated different domains of burden, including general self-experienced and self-reported well-being, mental and physical health, daily and social activities and on household members, work and individual protective measures.

The limitations of our study included firstly that even though we were able to include a heterogeneous group of severely immunocompromised individuals, they may not be fully representative of the entire severely immunocompromised population in The Netherlands. The distribution of reasons for or types of a severely impaired immune system may not be fully representative of the total severely immunocompromised population (RIVM, 2023d), and given the size of our survey we likely have missed less common conditions and immunosuppressive treatments. Secondly, we recruited participants via patient organizations – thereby relying on the contact-lists of their members. This may have led to a bias in our sample, e.g., potentially more involved or concerned individuals who were willing to participate, and therefore may have been less representative of the overall immunocompromised population in The Netherlands. Thirdly, we relied on entirely self-reported data for all of our measures, including reasons for being severely immunocompromised, which we did not verify through validation studies or reviews medical records. The respondents were asked to retrospectively answer questions during survey conduct about their perceptions and behaviors for the time before onset of the COVID-19 pandemic (i.e., at the end of 2019). This may have resulted in recall bias, although it is not certain in which direction this would have biased the results. Comparisons of responses between the time before the pandemic with the current situation should therefore be made with this in mind. Finally, due to the cross-sectional, non-interventional design of our study and self-reported data, we could not formally conclude that COVID-19 avoidance behaviors impacted or caused the various measures of self-experienced burden. However, our findings provide reasonable evidence that such avoidance behaviors may have impacted or caused the reported burden.

Almost all respondents reported that they had been vaccinated against COVID-19, but further details, including how many (booster) vaccinations had been received, were not within the scope of our study.

The survey responses were obtained from the end of May until early August 2023. Due to seasonal variations in viral circulation, this is a period when individuals would typically have had a lower risk of contracting COVID-19 than in the autumn and winter months. Also, due to the weather in this period, outdoor social activities would have been easier to organize. Therefore, the answers at the time of completing the survey may not be representative of the situation in other times of the year (e.g., autumn and winter).

Despite some limitations, our results indicate that the fact that, in the context of COVID-19 still circulating and causing hospitalizations and mortality, people who are severely immunocompromised in The Netherlands continue to have a self-experienced burden while avoiding COVID-19. Even in the absence of government-imposed societal measures for COVID-19 control, severely immunocompromised individuals have a decreased general well-being. Especially for the 1 out of 4 severely immunocompromised individuals in our study who were found to be shielding themselves during survey conduct, their self-experienced burden is most pronounced. These results indicate that specific, effective preventive measures and/or care are needed for this vulnerable group in addition to the national booster COVID-19 vaccination program.

## Funding

AstraZeneca BV provided funding to Motivaction BV for the conduct of the survey in our study. The institution of Chantal P. Rovers (Radboud university medical center) received consultancy fees from AstraZeneca for her services for our study. At the time of the study design, conduct and reporting, Jan Pander, Wendy Beekman-Hendriks and Neeltje Coolen were employees of AstraZeneca.

## CRediT authorship contribution statement

**Jan Pander:** Writing – review & editing, Writing – original draft, Visualization, Supervision, Methodology, Formal analysis, Conceptualization. **Wendy Beekman-Hendriks:** Writing – review & editing, Supervision, Project administration, Methodology, Conceptualization. **Neeltje Coolen:** Writing – review & editing, Visualization, Methodology, Conceptualization. **Valerie van de Flier:** Writing – review & editing, Validation, Investigation, Conceptualization. **Jeroen Senster:** Writing – review & editing, Software, Investigation, Formal analysis, Data curation. **Chantal P. Rovers:** Writing – review & editing, Validation, Supervision, Conceptualization.

## Declaration of competing interest

The authors declare that they have no known competing financial interests or personal relationships that could have appeared to influence the work reported in this paper.

## Data Availability

Data will be made available on request.

## References

[b0005] Antoun J., Brown D.J., Jones D.J.W., Sangala N.C., Lewis R.J., Shepherd A.I., Mcnarry M.A., Mackintosh K.A., Mason L., Corbett J., Saynor Z.L. (2021). Understanding the impact of initial COVID-19 restrictions on physical activity, wellbeing and quality of life in shielding adults with end-stage renal disease in the United Kingdom dialysing at home versus in-centre and their experiences with telemedicine. Int. J. Environ. Res. Public Health.

[b0010] Bernardi, L., Daniels, J. 2023. *Forsaken but engaged: An inquiry into the psychological aspects of COVID-19, mental health, and political engagement of immunocompromised people.* [Online]. Available: https://www.liverpool.ac.uk/humanities-and-social-sciences/research/coronavirus-research/forgotten-forsaken/ [Accessed December 13, 2023].

[b0015] DUTCH FEDERATION FOR NEPHROLOGY. 2021. *Kernboodschap RIVM 3e vaccinatie immuungecompromitteerden* [Online]. Available: https://www.nefro.nl/nieuws/kernboodschap-rivm-3e-vaccinatie-immuungecompromitteerden [Accessed November 6th, 2023].

[b0020] DUTCH GOVERNMENT. 2023. *Kamerbrief over besluiten resterende adviezen COVID-19.* [Online]. Available: https://www.rijksoverheid.nl/documenten/kamerstukken/2023/03/10/kamerbrief-over-besluiten-resterende-adviezen-covid-19 [Accessed November 6th, 2023].

[b0025] Evans R.A., Dube S., Lu Y., Yates M., Arnetorp S., Barnes E., Bell S., Carty L., Evans K., Graham S., Justo N., Moss P., Venkatesan S., Yokota R., Ferreira C., Mcnulty R., Taylor S., Quint J.K. (2023). Impact of COVID-19 on immunocompromised populations during the Omicron era: Insights from the observational population-based INFORM study. Lancet Reg. Health Eur..

[b0030] Hyams C., Challen R., Marlow R., Nguyen J., Begier E., Southern J., King J., Morley A., Kinney J., Clout M., Oliver J., Gray S., Ellsbury G., Maskell N., Jodar L., Gessner B., McLaughlin J., Danon L., Finn A., Avon C.A.P.R.G. (2023). Severity of Omicron (B.1.1.529) and Delta (B.1.617.2) SARS-CoV-2 infection among hospitalised adults: A prospective cohort study in Bristol, United Kingdom. Lancet Reg. Health Eur..

[b0035] Jassat W., Abdool Karim S.S., Mudara C., Welch R., Ozougwu L., Groome M.J., Govender N., Von Gottberg A., Wolter N., Wolmarans M., Rousseau P., Group D.A., Blumberg L., Cohen C. (2022). Clinical severity of COVID-19 in patients admitted to hospital during the omicron wave in South Africa: A retrospective observational study. Lancet Glob. Health.

[b0040] Lee A., Wong S.Y., Chai L.Y.A., Lee S.C., Lee M.X., Muthiah M.D., Tay S.H., Teo C.B., Tan B.K.J., Chan Y.H., Sundar R., Soon Y.Y. (2022). Efficacy of covid-19 vaccines in immunocompromised patients: systematic review and meta-analysis. BMJ.

[b0045] Lloyd A., Arnetorp S., Marcus J., Yokota R.T.C., Herring T.A., Powell P.A., Rohay J., Severens J.L., Venkatesan S., Williams P., Maia T., Taylor S., Krol M., Ware Jr J.E. (2023). Health Related quality of life and health utility values across different levels of physically distancing behaviors to avoid COVID-19 in immunocompromised adults - The eagle study. Value Health.

[b0050] Powell P.A., Williams P., Herring T.A., Venkatesan S., Arnetorp S., Lloyd A., Marcus J., Ouwens M., Rohay J., Severens J.L., Yokota R., Maia T., Taylor S., Krol M., Ware Jr J.E. (2023). Physical distancing to avoid COVID-19 and the association with humanistic burden in immunocompromised adults – The eagle study. Value Health.

[b0055] RIVM. 2022a. *Gedragswetenschappelijk onderzoek COVID-19. Resultaten 21e ronde: Welbevinden en leefstijl* [Online]. Available: https://www.rivm.nl/gedragsonderzoek/maatregelen-welbevinden/resultaten-21e-ronde/welbevinden-en-leefstijl [Accessed November 16, 2023].

[b0060] RIVM. 2022b. *Tijdlijn van coronamaatregelen* [Online]. Available: https://www.rivm.nl/gedragsonderzoek/tijdlijn-van-coronamaatregelen-2020 [Accessed March 10th, 2023].

[b0065] RIVM. 2023a. *Deelname COVID-19-vaccinatie in Nederland* [Online]. Available: https://www.rivm.nl/sites/default/files/2023-05/Deelname-COVID-19-vaccinatie-in-Nederland_20230508_def.pdf [Accessed November 6th, 2023].

[b0070] RIVM. 2023b. *Gedragswetenschappelijk onderzoek COVID-19. Naleving van en draagvlak voor de basis gedragsregels.* [Online]. Available: https://www.rivm.nl/gedragsonderzoek/trendonderzoek [Accessed November 16, 2023].

[b0075] RIVM. 2023c. *Handleiding COVID-19-vaccinatie van immuungecompromitteerde patiënten, version 27 Feb 2023.* [Online]. Available: https://lci.rivm.nl/handleiding-covid-19-vaccinatie-van-immuungecompromitteerde-patienten [Accessed March 10th, 2023].

[b0080] RIVM. 2023d. *Risicogroepen en COVID-19* [Online]. Available: https://www.rivm.nl/corona/covid-19/risicogroepen [Accessed].

[b0085] Shoham S., Batista C., Ben Amor Y., Ergonul O., Hassanain M., Hotez P., Kang G., Kim J.H., Lall B., Larson H.J., Naniche D., Sheahan T., Strub-Wourgaft N., Sow S.O., Wilder-SMITH A., Yadav P., Bottazzi M.E., Lancet Commission On C.-V., Therapeutics Task F. (2023). Vaccines and therapeutics for immunocompromised patients with COVID-19. EClinicalMedicine.

[b0090] Tartof S.Y., Slezak J.M., Puzniak L., Hong V., Xie F., Ackerson B.K., Valluri S.R., Jodar L., McLaughlin J.M. (2022). Immunocompromise and durability of BNT162b2 vaccine against severe outcomes due to omicron and delta variants. Lancet Respir. Med..

[b0095] Westcott K.A., Wilkins F., Chancellor A., Anderson A., Doe S., Echevarria C., Bourke S.J. (2021). The impact of COVID-19 shielding on the wellbeing, mental health and treatment adherence of adults with cystic fibrosis. Future Healthc. J..

[b0100] WHO. 2023a. *Coronavirus disease (COVID-19) pandemic* [Online]. Available: https://www.who.int/europe/emergencies/situations/covid-19 [Accessed March 10th, 2023].

[b0105] WHO. 2023b. *Statement on the fifteenth meeting of the IHR (2005) Emergency Committee on the COVID-19 pandemic (May 5th, 2023)* [Online]. Available: https://www.who.int/news/item/05-05-2023-statement-on-the-fifteenth-meeting-of-the-international-health-regulations-(2005)-emergency-committee-regarding-the-coronavirus-disease-(covid-19)-pandemic [Accessed November 16th, 2023].

[b0110] WHO. 2024. *COVID-19 Epidemiological Update (Edition 165, Published 15 March 2024)* [Online]. Available: https://www.who.int/publications/m/item/covid-19-epidemiological-update-15-march-2024 [Accessed March 18th, 2024].

